# Heritability of R2* iron in the basal ganglia and cortex

**DOI:** 10.18632/aging.204212

**Published:** 2022-08-09

**Authors:** Edith Hofer, Lukas Pirpamer, Christian Langkammer, Christian Tinauer, Sudha Seshadri, Helena Schmidt, Reinhold Schmidt

**Affiliations:** 1 Division of Neurogeriatrics, Department of Neurology, Medical University of Graz, Styria, Austria; 2 Institute for Medical Informatics, Statistics and Documentation, Medical University of Graz, Styria, Austria; 3 Department of Neurology, Medical University of Graz, Styria, Austria; 4 Glenn Biggs Institute for Alzheimer's and Neurodegenerative Diseases, University of Texas Health Sciences Center, San Antonio, TX 78229, USA; 5 Department of Neurology, Boston University School of Medicine, Boston, MA 02118, USA; 6 Research Unit-Genetic Epidemiology, Gottfried Schatz Research Centre for Cell Signalling, Metabolism and Aging, Molecular Biology and Biochemistry, Medical University of Graz, Graz, Styria, Austria

**Keywords:** brain iron, heritability, magnetic resonance imaging, genetic correlation, genetic and environmental factors

## Abstract

Background: While iron is essential for normal brain functioning, elevated concentrations are commonly found in neurodegenerative diseases and are associated with impaired cognition and neurological deficits. Currently, only little is known about genetic and environmental factors that influence brain iron concentrations.

Methods: Heritability and bivariate heritability of regional brain iron concentrations, assessed by R2* relaxometry at 3 Tesla MRI, were estimated with variance components models in 130 middle-aged to elderly participants of the Austrian Stroke Prevention Family Study.

Results: Heritability of R2* iron ranged from 0.46 to 0.82 in basal ganglia and from 0.65 to 0.76 in cortical lobes. Age and BMI explained up to 12% and 9% of the variance of R2* iron, while APOE ε4 carrier status, hypertension, diabetes, hypercholesterolemia, sex and smoking explained 5% or less. The genetic correlation of R2* iron among basal ganglionic nuclei and among cortical lobes ranged from 0.78 to 0.87 and from 0.65 to 0.97, respectively. R2* rates in basal ganglia and cortex were not genetically correlated.

Conclusions: Regional brain iron concentrations are mainly driven by genetic factors while environmental factors contribute to a certain extent. Brain iron levels in the basal ganglia and cortex are controlled by distinct sets of genes.

## INTRODUCTION

Iron is vital for normal brain function but iron excess can cause cell damaging oxidative stress [1, 2]. Brain iron, especially in the basal ganglia, accumulates with age until the 4th decade of life and plateaus afterwards [3, 4]. Elevated brain iron levels have also been observed in neurodegenerative diseases, including Parkinson‘s disease (PD) and Alzheimer’s disease (AD) [1, 5, 6], and are associated with decreased cognitive performance in elderly individuals [7–11].

Currently, only little is known about genetic and environmental factors that influence brain iron concentrations. Known genetic factors include iron metabolism genes, such as the hemochromatosis gene (HFE) and the transferrin gene (TF) [12–17]. A genome-wide association study in the UK Biobank has identified additional genes and has estimated the heritability of brain iron in deep grey matter structures to range between 0.08 and 0.58 [18]. Some studies indicate an interaction with the APOE gene, whose ε4 allele is the major risk factor for AD [19–24]. BMI, diabetes, hypertension and smoking have been identified as lifestyle factors associated with brain iron [10, 25–30]. An association with hypercholesterolemia, another potential brain iron modulating factor, has not yet been confirmed [26, 31]. Furthermore, it is not yet known whether brain iron in the basal ganglia and in the cortex are influenced by the same genes.

In the current study we investigated the amount of genetic and non-genetic contribution to brain iron by determining the heritability (h^2^) which represents the phenotypic variance attributable to genetic effects. We quantified brain iron by magnetic resonance imaging (MRI) based R2* relaxometry, which was shown to strongly correlate with the mass spectrometry measured absolute iron content in post-mortem studies [4, 32, 33]. For R2* iron in basal ganglia and in the cortex we estimated first, the heritability, second, the amount of variance that was explained by APOE ε4 carrier status, BMI, diabetes, hypertension, hypercholesterolemia and smoking and third, the genetic correlation between brain regions. Data are from the Austrian Stroke Prevention Family Study (ASPS-Fam).

## RESULTS

### Sample characteristics

The study sample includes 130 individuals from 59 families. Of these families, 47 comprise 2 members and 12 comprise 3 members. The family pedigrees consist of 55 sibling pairs, 19 parent-child pairs, 8 avuncular pairs and one pair of half-siblings. The median age is 72 [interquartile range (IQR): 61-75], ranging from 38 to 85 years. The study includes 71 (54.6%) females and 22 (16.9%) participants carry at least one APOE ε4 allele. Mean and standard deviation of the BMI are 25.6 +- 3.9 and 105 (80.8%), 13 (10%) and 110 (84.6%) individuals suffer from hypertension, diabetes and hypercholesterolemia respectively. Current or former smokers make up 45.4% of the study population (59 individuals).

### Heritability

**Figure 1:**
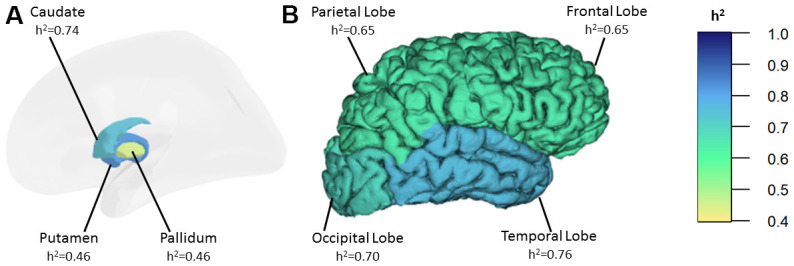
Heritability estimates of R2* iron in the basal ganglia (**A**) and in the cortex (**B**). h^2^: heritability.

Univariate heritability estimates of R2* iron in the basal ganglia and in the cortex are presented in Table 1 and Figure 1. In the basic model, adjusting for age and sex, the heritability of R2* iron in the basal ganglia (h^2^=0.75, p=0.001) is similar to the cortex (h^2^=0.73, p=0.001). The heritability is highest in the putamen (h^2^=0.82, p=0.001) and the caudate (h^2^=0.74, p=0.001) followed by the pallidum (h^2^=0.46, p=0.025). In the cortex, we found the highest heritability in the temporal lobe (h^2^=0.76 p=0.001) and the lowest heritability in the frontal (h^2^=0.65 p=0.003) and the parietal lobe (h^2^=0.65 p=0.002). The variance in R2* iron due to age ranged from 1% to 12%, while sex explained only 2% of the variance of R2* iron in the frontal lobe.

**Table 1:** Heritability of R2* iron in the basal ganglia and cortex (N=130).

	**h^2^**	**SE**	**p**	**Final covariates**	**Proportion of variance due to all final covariates**
**Basal Ganglia**	0.75	0.20	0.001	age	0.10
Caudate	0.74	0.20	0.001	age	0.09
Pallidum	0.46	0.22	0.025	-	
Putamen	0.82	0.19	0.001	age	0.12
**Total Cortex**	0.73	0.21	0.001	-	
Frontal Lobe	0.65	0.22	0.003	sex	0.02
Temporal Lobe	0.76	0.21	0.001	age	0.04
Parietal Lobe	0.65	0.22	0.002	age	0.01
Occipital Lobe	0.70	0.22	0.002	age	0.05

Heritability estimates were obtained from the basic model with covariate screening for age and sex.

N: sample size, h^2^: heritability, SE: standard error, p: false discovery rate corrected p-value.

### Proportion of variance in R2* brain iron due to genetic and risk factors

APOE ε4 carrier status explained up to 5% of the phenotypic variance in the basal ganglia (Table 2). The effect of BMI was significant in all regions and explained up to 4% and 9% of the variance of R2* iron in the basal ganglia and cortical lobes, respectively. Hypertension explained up to 4% of R2* variance in the cortex, while diabetes explained up to 4% in the basal ganglia. Hypercholesterolemia explained 1% and 2% of the R2* variance in the parietal and temporal lobe respectively. The effect of smoking status was not significant in any of the regions. The direction of the association between significant risk factors and R2* rates is positive, indicating higher R2* rates in the presence of these factors (Table 2). Conversely, APOE ε4 carrier status is negatively associated with R2* rates.

**Table 2:** Proportion of variance in R2* brain iron due to genetic and environmental factors (N=130).

	Proportion of variance of R2* brain iron due to				
	**APOE ε4 carrier status**	**BMI**	**Hypertension**	**Diabetes**	**Hypercholesterolemia**
**Basal Ganglia**	0.05 (-)	0.01 (+)		0.02 (+)	
Caudate	0.04 (-)	0.04 (+)		0.03 (+)	
Pallidum	0.03 (-)	0.02 (+)		0.04 (+)	
Putamen	0.05 (-)	0.01 (+)		0.01 (+)	
**Total Cortex**		0.06 (+)	0.04 (+)		
Frontal Lobe		0.06 (+)			
Temporal Lobe		0.09 (+)	0.03 (+)	0.00 (+)	0.02 (+)
Parietal Lobe		0.03 (+)	0.04 (+)		0.01 (+)
Occipital Lobe		0.02 (+)	0.03 (+)		

### Bivariate heritability analyses

**Figure 2:**
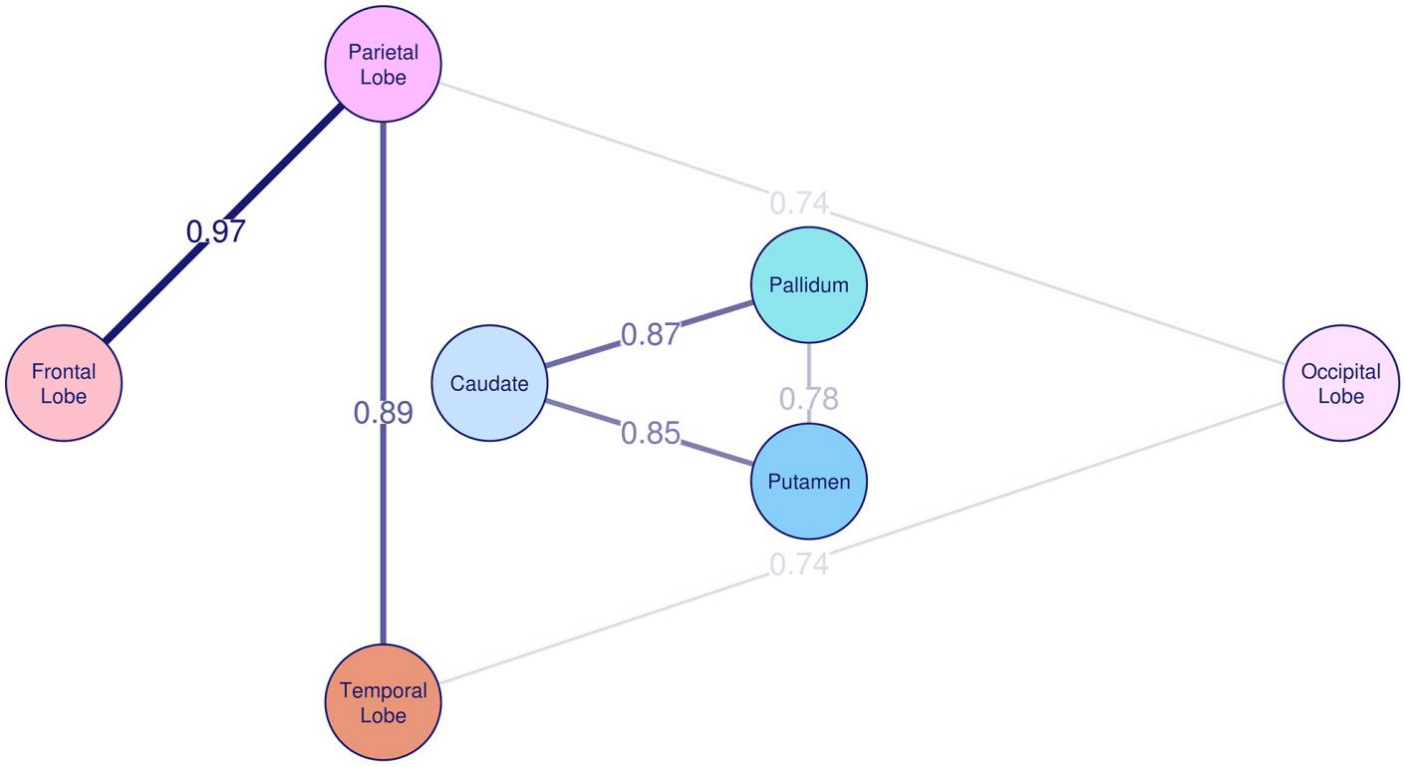
Genetic correlation of R2* among basal ganglia and cortex. The genetic correlation coefficient r_g_ is plotted on the lines and also represented by the thickness of the lines between the brain regions.

The correlation of R2* rates between the different brain regions is summarized in Table 3. The SOLAR estimated phenotypic correlation among basal ganglia, ranging from 0.55 to 0.84, and among lobes, ranging from 0.59 to 0.81, was significant. Between basal ganglia and lobes, the correlation of R2* rates was weak and ranged between 0.09 and 0.29. Spearman correlation analysis yielded similar results. Table 4 and Figure 2 show strong and significant genetic correlation of R2* rates among individual basal ganglia structures, with the genetic correlation coefficient (r_g_) between 0.78 and 0.87, and between lobes, with r_g_ between 0.68 and 0.97. The genetic correlation between frontal and occipital lobe was borderline significant with r_g_=0.65 and p=0.08. We did not find significant genetic correlation between basal ganglia and cortical lobe R2* rates, and neither environmental correlations between any of the investigated brain regions.

**Table 3:** Phenotypic correlation of R2* iron in the basal ganglia and cortex (N=130).

	**Caudate**	**Pallidum**	**Putamen**	**Frontal lobe**	**Temporal lobe**	**Parietal lobe**	**Occipital lobe**
**Caudate**		**0.55** **(p=1.91E-10)**	**0.84** **(p=5.06E-25)**	**0.27** **(p=7.75E-03)**	**0.22** **(p=3.61E-02)**	**0.24** **(p=1.58E-02)**	**0.25** **(p=1.29E-02)**
**Pallidum**	**0.53** **(p=2.63E-10)**		**0.63** **(p=6.06E-13)**	**0.24** **(p=1.19E-02)**	0.09 (p=3.12E-01)	0.11 (p=2.29E-01)	**0.29** **(p=3.53E-03)**
**Putamen**	**0.88** **(p=6.45E-42)**	**0.56** **(p=9.00E-12)**		**0.26** **(p=1.15E-02)**	0.11 (p=3.12E-01)	0.16 (p=1.01E-01)	**0.25** **(p=1.27E-02)**
**Frontal Lobe**	**0.23** **(p=1.32E-02)**	**0.23** **(p=1.37E-02)**	**0.22** **(p=1.45E-02)**		**0.59** **(p=1.51E-10)**	**0.81** **(p=9.97E-25)**	**0.68** **(p=1.90E-13)**
**Temporal Lobe**	**0.27** **(p=3.47E-03)**	0.06 (p=5.30E-01)	0.18 (p=5.67E-02)	**0.51** **(p=1.12E-09)**		**0.72** **(p=1.39E-16)**	**0.66** **(p=3.18E-13)**
**Parietal Lobe**	0.13 (p=1.67E-01)	0.12 (p=1.78E-01)	0.08 (p=3.84E-01)	**0.81** **(p=6.02E-30)**	**0.64** **(p=6.72E-16)**		**0.69** **(p=9.24E-15)**
**Occipital Lobe**	**0.30** **(p=9.79E-04)**	**0.27** **(p=2.78E-03)**	**0.30** **(p=1.16E-03)**	**0.61** **(p=4.76E-14)**	**0.65** **(p=6.72E-16)**	**0.61** **(p=3.82E-14)**	

Upper diagonal: phenotypic correlation estimated by SOLAR software; lower diagonal: phenotypic correlation estimated by Spearman’s correlation coefficient; N: sample size; p: false discovery rate corrected p-value; in bold: false discovery rate corrected p-value < 0.05.

**Table 4:** Genetic and environmental correlation of R2* in the basal ganglia and cortex (N=130).

	**Caudate**	**Pallidum**	**Putamen**	**Frontal lobe**	**Temporal lobe**	**Parietal lobe**	**Occipital lobe**
**Caudate**		**0.87±0.20 (p=0.04)**	**0.85±0.07 (p=0.02)**	0.04±0.26 (p=0.93)	-0.06±0.23 (p=0.93)	-0.05±0.27 (p=0.93)	0.02±0.26 (p=0.93)
**Pallidum**	0.16±0.37 (p=0.74)		**0.78±0.16 (p=0.04)**	0.10±0.33 (p=0.93)	0.03±0.32 (p=0.93)	0.15±0.32 (p=0.93)	0.23±0.31 (p=0.86)
**Putamen**	0.83±0.20 (p=0.54)	0.49±0.32 (p=0.54)		0.20±0.24 (p=0.84)	-0.17±0.22 (p=0.84)	0.04±0.25 (p=0.93)	0.10±0.24 (p=0.93)
**Frontal Lobe**	0.84±0.44 (p=0.46)	0.43±0.31 (p=0.54)	0.46±0.52 (p=0.57)		**0.68±0.17 (p=0.04)**	**0.97±0.07 (p=0.02)**	0.65±0.16 (p=0.08)
**Temporal Lobe**	1.00^a^ (p=0.42)	0.20±0.38 (p=0.68)	1.00^a^ (p=0.42)	0.41±0.38 (p=0.57)		**0.89±0.11 (p=0.02)**	**0.74±0.14 (p=0.04)**
**Parietal Lobe**	0.93±0.46 (p=0.42)	0.08±0.35 (p=0.82)	0.55±0.56 (p=0.54)	0.51±0.29 (p=0.54)	0.33±0.41 (p=0.66)		**0.74±0.13 (p=0.04)**
**Occipital Lobe**	0.86±0.51 (p=0.46)	0.38±0.33 (p=0.54)	0.76±0.58 (p=0.54)	0.74±0.23 (p=0.54)	0.48±0.36 (p=0.57)	0.60±0.27 (p=0.54)	

Upper diagonal: genetic correlation r_g_ ± standard error estimated by SOLAR software; lower diagonal: environmental correlation r_e_ ± standard error estimated by SOLAR software.

^a^an estimate of the environmental correlation at this boundary of 1.0 is not precise, as no standard error can be obtained by the SOLAR software in this case.

N: sample size; p: false discovery rate corrected p-value; in bold: false discovery rate corrected p-value < 0.05.

## DISCUSSION

Here we show that the heritability of R2* iron in basal ganglia and cortex in a general population of middle-aged to elderly individuals was moderate to high, with estimates ranging from 0.46 to 0.82. While age and BMI explained up to 12% and 9% of the variance in brain iron respectively, APOE ε4 carrier status, hypertension, diabetes, hypercholesterolemia and sex explained 5% or less, and smoking status did not explain any variance. We did not observe significant genetic correlations between R2* iron concentrations in basal ganglia and cortical lobes, but strong positive genetic correlation among cortical lobes and among basal ganglionic nuclei.

In all investigated regions, except for the pallidum, two-thirds or more of the variance in R2* iron can be explained by genetic factors. Our heritability estimates for R2* iron in the basal ganglia (h^2^_caudate_=0.74, h^2^_pallidum_=0.46, h^2^_putamen_=0.82) are higher than those from the UK Biobank [18] (h^2^_left caudate_=0.38, h^2^_right caudate_=0.37; h^2^_left pallidum_=0.50, h^2^_right pallidum_=0.46; h^2^_left putamen_=0.50, h^2^_right putamen_=0.58), except for the pallidum. This discrepancy might be explained by the different heritability estimation methods: pedigree-based heritability in ASPS-Fam compared to heritability based on common single nucleotide polymorphisms (SNPs) in the UK Biobank [34]. Nevertheless, in both studies, the highest heritability was found in putamen.

While heritability provides an estimate of the variance in a trait that is determined by genetics, it does not reveal gene-specific influences. Therefore, we added the APOE ε4 carrier status as a covariate to the heritability model to estimate how much of the variance in brain iron is attributable to this allele. We found that the APOE ε4 carrier status explains between 3% and 5% of the variance of R2* iron in the basal ganglia, corroborating previous findings which indicate that the APOE gene influences brain iron levels [19, 22, 26]. This is of particular interest as the basal ganglia are the brain structures with the highest iron concentrations in healthy brains [1, 32], and abnormally increased basal ganglia iron levels are often found in neurodegenerative diseases [1, 6, 35], for which the APOE ε4 allele is a major risk factor. Moreover, APOE seems to interact with iron homeostasis genes like HFR in individuals with cognitive impairment [19] and Kargerer et al. [36] found that APOE4 moderates the effects of cortical iron on brain function in healthy elderly. The association between APOE ε4 carrier status and brain iron concentrations in basal ganglia in our study was negative meaning that APOE ε4 carriers had lower measured iron Data indicate significant (p<0.1) covariates in corresponding model. The direction of the association between the covariates and the R2* rates is provided in brackets. N: sample size.

deposition than non-carriers. APOE ε4 is considered a major risk factor for AD und thus one might have rather assumed a positive relationship. Nonetheless, the effect of APOE ε4 on R2* brain iron concentrations might not be apparent until the age that people are at risk for AD (> 65 years), when APOE ε4 begins to influence risk. The lack of association in our cohort might therefore be related to the low number of APOE ε4 carriers who were older than 65 years. However, in line with our study findings, a lack of association between APOE ε4 brain iron levels was also reported in the Memory and Aging Project, which is a clinical-neuropathological cohort study of older adults [37].

The moderate to high phenotypic correlation of R2* brain concentrations among basal ganglia and among cortical lobes can be mainly attributed to genetic correlation. This reflects that the variance of brain iron in basal ganglia and in cortex is influenced to a large extent by the same genes, but due to the missing genetic correlation between basal ganglia and cortex, these sets of genes appear to be distinct from one another.

Heritability estimates between 0.46 and 0.82 in our study imply that between 54% and 18% of the variance in brain iron can be explained by environmental factors. In our study, BMI had an effect on the variance of brain iron which confirms previous findings in a partly overlapping cohort [25]. This result is supported by other studies that linked obesity to elevated brain iron levels in human [10, 38] and in mice [39]. We also confirmed that diabetes had an effect on the variance of iron in basal ganglia [26, 27, 29]. In line with Li et al. [26], hypercholesterolemia lacked effects on iron in basal ganglia in the current study, but it explained two and one percent of the iron in the temporal and parietal lobe. One possible explanation for these effects on brain iron is that the APOE ε4 allele, obesity, insulin resistance and elevated blood lipid levels have been previously linked to an increased permeability of the blood-brain barrier [31, 40–42], which is a possible cause for subsequent brain iron accumulation [1, 43–45]. Hypertension explained 3% of the variance of iron in the temporal and occipital lobe consistently with Rodrigue et al. [30] who found elevated iron levels in the entorhinal cortex and the primary visual cortex in hypertensives. In accordance with Li et al. [26], hypertension had no effect on the variance of basal ganglia iron in our study, although Rodrigue et al. [30] found an effect in caudate and putamen. While smoking was associated with brain iron in deep grey matter in a previous study on ASPS-Fam data [25] and in thalamus in a recent study [26], we found no effect. A reason for these contradicting results may be the different definitions of the lifestyle factors, sample sizes, regions examined and methods used for brain iron quantification in these studies. We did not find evidence for shared environmental effects among and between basal ganglia and lobes, which suggests that environmental factors that affect the variation of R2* brain iron in different brain regions are independent from each other. In line with previous studies [1, 3, 26, 30], age explained with up to 12% the largest amount of the variance in brain iron.

A limitation of this study is the rather small sample size which may prevent findings with smaller effect sizes. Moreover, heritability provides an estimate on the amount of variance in R2* brain iron that is determined by genetics but it does not allow the detection of specific genetic loci. Such loci could be identified by genome wide association studies, which unbiasedly screen the whole genome for genetic variants but also require very large sample sizes. A lack of genetic data for most of the study participants prevented us from investigating other genetic factors than APOE, such as iron metabolism-related genes like HFE and TF. Another limitation is the restricted battery of environmental factors examined. For instance, no information about diet or iron intake is available in this cohort but brain iron levels seem to be influenced by diet [46, 47]. Furthermore, it is important to note that R2* rates are not only sensitive to brain iron, but also to the myelin content [48, 49], even though iron is a stronger contributor to R2* rates in gray matter areas [50], which are the focus of the present study.

## CONCLUSIONS

Our results demonstrate that brain iron accumulation is a complex and multi-factorial trait that is influenced by both, genetic and environmental, determinants. Although several genes that modulate brain iron levels are already known, further genetic studies may reveal additional genes, especially as it seems that brain iron concentration in the basal ganglia and cortex is influenced by distinct sets of genes. Concurrently, it would be interesting to examine potential environmental risk factors more closely in future studies.

## MATERIALS AND METHODS

### Study sample

The ASPS-Fam is a prospective single-center community-based study on the cerebral effects of vascular risk factors in residents of the city of Graz, Austria, without clinical signs and symptoms of stroke and dementia and a normal neurological examination [7, 25]. The ASPS-Fam is an extension of the Austrian Stroke Prevention Study (ASPS), which was established in 1991 [51, 52]. Between 2006 and 2013, study participants of the ASPS and their first-degree relatives were invited to join the ASPS-Fam. A total of 419 individuals from 176 families were included in the study. The number of members per family ranged from 2 to 6. The entire cohort underwent an extended diagnostic work-up including clinical history, blood tests, cognitive testing, vascular risk factor assessment and brain MRI. We included those 130 participants with complete brain 3 Tesla MRI, laboratory, and risk factor data who had at least one family member with these data available. The study protocol was approved by the ethics committee of the Medical University of Graz, Austria, and written informed consent was obtained from all participants.

### Magnetic resonance imaging

MRI scans were obtained from a 3 Tesla scanner (Magnetom TrioTim; Siemens Healthcare, Erlangen, Germany) with a 12-channel head coil. The MRI study protocol included a T1-weighted 3D sequence with magnetization prepared raid gradient echo (MPRAGE) with whole brain coverage, 1mm isotropic resolution, 1900 ms repetition time, 2.19 ms echo time, 900 ms inversion time and 9° flip angle for subsequent automated tissue segmentation. R2* relaxation data were acquired using a spoiled 3D multi-echo gradient echo sequence (FLASH) with 1x1x2 mm^3^ resolution, 64 slices, 35 ms repetition time, 15° flip angle and 6 equally spaced echoes from 4.92 ms echo time with 4.92 ms echo spacing. R2* maps were processed by an in house developed optimized fitting algorithm [53], which takes the noise of each echo into account and is freely available online (https://github.com/neuroimaging-mug/relaxometry). A semiquantitative T2-map was assessed by a T2-weighted sequence with 2 echoes (TE1/TE2/TR =10/72/5260 ms, 40 slices and with 0.86x0.86x3 mm^3^ resolution). For assessment of regional R2*, the FreeSurfer toolset (version 5.3, 2017, http://surfer.nmr.mgh.harvard.edu) [54, 55] was used to automatically segment the cortex and basal ganglia [56, 57]. The segmentations included the global cortex, frontal, parietal, temporal and occipital lobes, global basal ganglia, caudate nucleus, globus pallidus and putamen, separately for the left and the right hemisphere. Obtained segmentation masks were affinely registered to the gradient echo magnitude using FSL-flirt (FSL, version 6, http://fsl.fmrib.ox.ac.uk/fsl) and subsequently eroded to prevent partial volume effects. Further, to avoid CSF-contaminated voxels in the registered cortical masks, we performed a mask-segmentation optimization using a semi-quantitative T2-map, as previously described [58]. The median R2* values were calculated for the left and right hemisphere of each region, and the mean of the two hemispheres was used for the analyses.

### Risk factors

The APOE risk factor was included as APOE ε4 carriers versus non-carriers and smoking was defined as current or former smoker versus non-smoker. Hypertension was considered as history of hypertension and/or systolic blood pressure over 140 mmHg or a diastolic blood pressure over 90 mmHg and/or current use of antihypertensive agents [59]. Diabetes mellitus was present if an individual had a history of diabetes, used anti-diabetics or had a fasting blood glucose level above 126 mg/dl (7.0 mmol/l) at the time of examination [60]. Presence of hypercholesterolemia was confirmed if a participant had a history of hypercholesterolemia, was treated for hypercholesterolemia at the time of examination or if the total or LDL cholesterol was higher than 200 mg/dl or 130 mg/dl respectively.

### Statistical analysis

Descriptive Statistics were calculated with the SPSS software (version 25, SPSS Inc., Chicago, IL, USA). Heritability of R2* iron in the cortex and basal ganglia was estimated with variance components models as implemented in the SOLAR software (version 8.1.1, http://solar-eclipse-genetics.org). SOLAR decomposes the phenotypic variance (V_p_) of R2* iron into polygenic variance (V_g_) and environmental variance (V_e_) [61]. V_p_ is the residual phenotypic variance which cannot be explained by the covariates. Covariates are modelled as fixed effects and the phenotype is residualized on these covariates. V_g_ is the variance due to additive effects of genes based on pedigree relationships. The pedigree structure is described by a kinship matrix which includes the amount of shared genes between each pair of individuals according to their relationship. For instance, sibling pairs and parent-child pairs share 50% of their genes while avuncular and half-sibling pairs share 25%. V_e_ is the variance due to environmental factors, the non-additive genetic components, and measurement errors. The variance components are estimated by comparing the observed phenotypic covariance matrix with the covariance matrix predicted by kinship, and the heritability is calculated as h^2^=V_g_/V_p_. Significance of the heritability is tested by comparing the likelihood of the model in which V_g_ is constrained to zero with that of a model in which V_g_ is estimated. The variance component models can be extended to bivariate analyses to determine if two phenotypes are influenced by the same genes. The phenotypic correlation (r_p_) between two quantitative traits can be partitioned into a genetic and an environmental component using the kinship information. Likelihood ratio tests are used to test if the genetic (r_g_) or the environmental (r_e_) correlation is different from zero.

We estimated the heritability of R2* iron in the basal ganglia and in the cortex using a basic model including age and sex as covariates. Extended models including age, sex and additionally either APOE ε4 carrier status, BMI, diabetes, hypertension, hypercholesterolemia or smoking were calculated to determine the proportion of variance in R2* iron due to lifestyle factors. Covariate screening was used to determine the statistical significance of each covariate effect and only significant covariates (p<0.1) were kept in the model. The significance of each covariate in the model was tested using a likelihood ratio test comparing the models with and without covariates. A liberal significance threshold of 0.1 was chosen to avoid the removal of relevant covariates from the model. The proportion of variance due to APOE ε4 carrier status, BMI, hypertension, diabetes, hypercholesterolemia or smoking was calculated by subtracting the proportion of variance due to all significant covariates from the model including the respective covariate from the proportion of variance due to covariates of the basic model. We also estimated the genetic correlation between R2* iron in the basal ganglia and cortical lobes with age and sex as fixed covariates. Since variance component methods as implemented in SOLAR are sensitive to deviations from the normal distribution [62], we applied rank-based inverse-normal transformation of the R2* values to ensure normal distribution. As we investigated 9 brain regions in total, we performed multiple testing correction separately for univariate heritability, Spearman correlation, SOLAR phenotypic correlation, SOLAR genetic correlation and SOLAR environmental correlation results using the false discovery rate (FDR) method [63]. The plots were computed using the packages ggseg3d (version 1.6.2, https://cran.rstudio.com/web/packages/ggseg3d/ggseg3d.pdf), fsbrain (version 0.4.3, https://cran.r-project.org/web/packages/fsbrain/vignettes/fsbrain.html) and qgraph (version 1.9.2, https://cran.r-project.org/web/packages/qgraph) in R (version 4.1.0, https://www.R-project.org).

### Data availability

Data sets generated and/or analyzed during the study are available from the corresponding author upon reasonable request.
